# Evidence for the intrinsically nonlinear nature of receptive fields in vision

**DOI:** 10.1038/s41598-020-73113-0

**Published:** 2020-10-01

**Authors:** Marcelo Bertalmío, Alex Gomez-Villa, Adrián Martín, Javier Vazquez-Corral, David Kane, Jesús Malo

**Affiliations:** 1 Universitat Pompeu Fabra, Barcelona, Spain; 2 Universitat de Valencia, Valencia, Spain

**Keywords:** Computational biology and bioinformatics, Neuroscience, Psychology

## Abstract

The responses of visual neurons, as well as visual perception phenomena in general, are highly *nonlinear* functions of the visual input, while most vision models are grounded on the notion of a *linear* receptive field (RF). The linear RF has a number of inherent problems: it changes with the input, it presupposes a set of basis functions for the visual system, and it conflicts with recent studies on dendritic computations. Here we propose to model the RF in a nonlinear manner, introducing the intrinsically nonlinear receptive field (INRF). Apart from being more physiologically plausible and embodying the efficient representation principle, the INRF has a key property of wide-ranging implications: for several vision science phenomena where a linear RF must vary with the input in order to predict responses, the INRF can remain constant under different stimuli. We also prove that Artificial Neural Networks with INRF modules instead of linear filters have a remarkably improved performance and better emulate basic human perception. Our results suggest a change of paradigm for vision science as well as for artificial intelligence.

## Introduction

In vision science, the receptive field (RF) of a neuron is the extent of the visual field where light influences the neuron’s response. In the “standard model” of vision the first stage is a filtering operation consisting of multiplying the intensities at each local region of an image stimulus by the values of a filter (the weights of the RF), and summing the resulting intensities^1^; this weighted sum may then be normalized by the responses of neighboring neurons and passed through a pointwise nonlinearity. Many scientists have come to accept this linear plus nonlinear (L+NL) formulation as a working model of the visual system^2^, and while there have been considerable improvements on, and extensions to, the standard model, the linear RF remains as the foundation of most vision models. The inspiration for Artificial Neural Networks (ANNs) came from early, classical models of biological neural networks, and for this reason they are also based on the linear RF, which is their cornerstone^3,4^.

But there are a number of problems that are inherent to considering the RF as having a linear form, of which we will highlight three.

### Adaptation makes the linear RF change with the input

For all species, adaptation is a key property that any neural system must have; in particular in the human visual system it is present in all stages, from the photoreceptors in the retina all the way to the cortex^5^. Adaptation constantly adjusts the sensitivity of the visual system to the properties of the stimulus, bringing the survival advantage of making perception approximately independent from lighting conditions while quite sensitive to small differences among neighboring regions^6,7^; this happens at very different timescales, from days and hours down to the 100ms interval between rapid eye movements, when retinal neurons adapt to the local mean and variance of the signal, approximating histogram equalization^8^. In this way, adaptation allows to encode neural signals with less redundancy, and is therefore an embodiment of the efficient representation principle^9,10^, an ecological approach for vision science that has proven to be extremely successful across mammalian, amphibian and insect species^11–14^ and that states that the organization of the visual system in general and neural responses in particular are tailored to the statistics of the images that the individual typically encounters, so that visual information can be encoded in the most efficient way, optimizing the limited biological resources.

Due to the fact that the visual system is nonlinear, it can be shown that the linear RF can’t be a fixed, constant property of a neuron^1,15,16^. It is visual adaptation which modifies the spatial receptive field and temporal integration properties of neurons depending on the input; in fact, under a basic L+NL formulation, adaptation simply means “a change in the parameters of the model”^17^.

The conclusion is that the RF of a neuron is not determined by its biological wiring but has different sizes, preferred orientations or even polarity (ON/OFF) for different stimuli^18–20^.

In vision models the RF is characterized by finding the linear filter that provides the best correlation between the visual input and some specific response data, be it psychophysical like brightness perception magnitude or neurophysiological like spike train timings, and for that data the model can normally achieve an excellent fit. But there is much evidence showing that model performance degrades quickly if any aspect of the stimulus, like the spatial frequency or the contrast, is changed^1,15^; the fact is that vision models and ANNs use RFs that are constant, or they don’t have general rules, valid for all inputs, as to how the RFs or the nonlinearities should be modified depending on the stimulus.

### The linear RF presupposes a set of basis functions for the visual system

The visual system is nonlinear, and therefore it has no basis functions. Vision scientists, although aware that neurons are nonlinear, model their responses by probing the visual system with a variety of mathematically elegant basis functions like spots, edges or gratings, which are ideal for analyzing linear systems but that for nonlinear systems have no particular meaning^2^; in fact, the estimated linear RF changes with the basis set used^21^. Models based on the linear RF are not tests of how well the RF describes the neuron’s behavior, they have been obtained simply by *assuming* that the neuron performs a linear summation followed by a nonlinearity and then searching for the best-fitting L+NL model.

Studying neurons using the complete set of possible stimuli, which according to the theory would be the only way to characterize the system with total confidence, is a task of impossibly high complexity, requiring in the order of $10^{1000}$ tests^2^. As experimental constraints impose to use just a tiny fraction of tests, the nonlinearities that are mapped are forced to be quite smooth. Therefore, nothing guarantees that the behavior of the visual system for all inputs, and in particular for complex stimuli as those given by natural scenes, can be characterized from studying a reduced set of stimuli.

### The linear RF is questioned by more recent works in vision science

Cortical neurons produce all-or-nothing action potentials instead of analog values, i.e. neurons are highly nonlinear, and more recent studies have rejected the hypothesis that individual neurons can be modeled as a linear RF followed by an output nonlinearity^22^, showing instead that each thin branch of the elaborate dendritic tree of a neuron could be computing many different nonlinear combinations of its inputs^2,23,24^.

### Weaknesses in the state-of-the-art

The linear RF limitations in predicting neuron responses to complex stimuli have been known for many years, and a wide variety of approaches have been introduced to model the nonlinear nature of visual phenomena, e.g. nonlinear subunits^25^, divisive normalization^26^, feedback connections^27^, neural-field equations^28^, nonlinear geometry^21^, nonlinear centre-surround interactions^29^, hierarchical models^30^, fitting ANNs to visual data^31^, or training ANNs to perform a high-level visual task^32^, to name some of the most relevant lines of research.

However, we must remark that all these approaches are still grounded in the notion of a linear RF. And what we currently have is that state-of-the-art vision models and ANNs, with their linear RFs, have very important weaknesses in their predictive powers.

In visual perception and color imaging, the general case of the image appearance problem is very much *open*: for natural images under given viewing conditions, there are neither fully effective automatic solutions nor accurate vision models to predict image appearance, not even in controlled scenarios like cinema theaters^8^. This is a very important topic for imaging technologies, which require good perception models in order to encode image information efficiently and without introducing visible artifacts, for proper color representation, processing and display.

In computer vision, some of the well-known and most relevant problems of ANNs can be described as a failure to emulate basic human perception abilities. For instance ANNs are prone to adversarial attacks, where a very small change in pixel values in an image of some object A can lead the neural network to misclassify it as being a picture of object B, while for a human observer both the original and the modified images are perceived as being identical^33^; this is a key limitation of ANNs, with an enormous potential for causing havoc. Another example is that the classification performance of ANNs falls rapidly when noise or texture changes are introduced on the test images, while human performance remains fairly stable under these modifications^34^. The difficulty of modeling vision with ANNs is a topic that is garnering increasing attention^35,36^.

In neuroscience, back in 2005 the standard model was able to explain at the most a $40\%$ of the data variance in V1^2^. That same year, Carandini et al.^1^ commented that the fact that the linear RF component per se, which they say is “the bread and butter of the standard model”, can explain just a small fraction of the response variance of cortical neurons, is a sobering realization that leaves little room for the optimistic hope that matters could be fixed adding more modulatory terms or nonlinearities. Confirming their assessment, fifteen years later the standard model (via a goal-driven deep ANN) is just able to explain around $50\%$ of the data variance in V1^37^.

Olshausen^38^ states that the problem is not in the lack of data but in the lack of a proper conceptual framework with which to analyze the data: the main obstacle is the standard model itself, which rather than be revised needs to be discarded altogether as it is the wrong starting point.

## Proposed approach: the intrinsically nonlinear receptive field (INRF)

In the classical model, the linear RF response at location *x* is expressed as a weighted sum of the input *I* at neighboring locations $y_i$:

1 $$\begin{aligned} LRF(x)=\sum _i w(x,y_i) I(y_i) \end{aligned}$$

More recent studies of single-neuron activity consider sigmoidal nonlinearities $\sigma (\cdot )$ at dendrites^22,39^, yielding a nonlinear summation model:

2 $$\begin{aligned} LNRF(x)=\sum _i w(x,y_i) \sigma (I(y_i)) \end{aligned}$$

We now remark the following three points about dendritic computations, that will be the basis of our proposed formulation:

1. Poirazi et al. mention that with the model of Eq. (2) predictions are far from perfect^39^, and suggest as a possible reason that a single nonlinearity $\sigma$ may not be adequate for all branches.
2. There are mechanisms that enable individual dendritic branches to act as nonlinear units^24^.
3. There is feedback from the neuron soma to the dendrites^23^.

In this paper we introduce the concept of an intrinsically nonlinear receptive field (INRF), a single-neuron summation model that we define in this way:

3 $$\begin{aligned} INRF(x)=\sum _i m_i I(y_i) -\lambda \sum _i w_i \sigma \left( I(y_i) - \sum _j g(y_j-x) I(y_j) \right) , \end{aligned}$$

where for simplicity we have used the notation $m_i=m(x,y_i), w_i=w(x,y_i)$.

**Figure 1:**
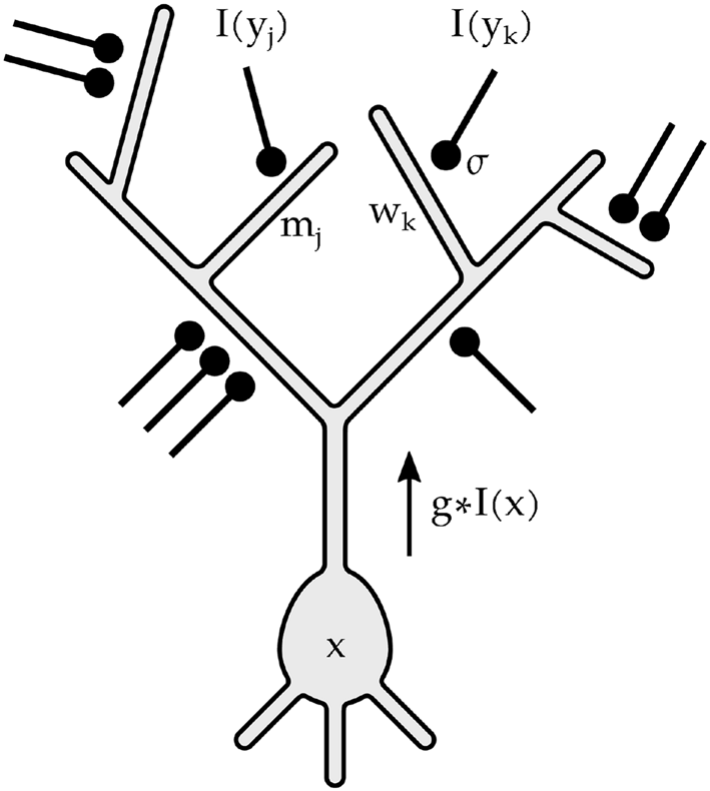
Schematic of single-neuron spatial summation with a INRF. Contributions of linear dendrites are summed with weights $m_i$. Contributions of nonlinear dendrites are summed with weights $w_i$. The nonlinearity $\sigma$ is shifted by a local average $g*I(x)$ of the signal *I* around point *x*, and this value is obtained by the nonlinear dendrites through feedback from the soma, represented by an arrow.

In the INRF, as schematized in Fig. 1, some dendrites are linear and their contributions are summed with weights $m_i$, while some other dendrites are nonlinear and their contributions are summed with weights $w_i$ (following points 1 and 2 above). The nonlinearity $\sigma$ is shifted by a local average of the signal around point *x*, given by the convolution with the kernel *g*, i.e. $\sum _j g(y_j-x) I(y_j) = g*I(x)$. This shift value could be obtained by the dendrites from feedback from the soma (following point 3 above), and is represented in Fig. 1 by an arrow.

Our INRF model is a model of *summation* alternative to the linear RF. The final neural output would be INRF followed by a nonlinearity such as rectification, divisive normalization^26^, etc.

## Properties of the INRF

### The INRF can’t be efficiently expressed in L+NL form

A key point about the INRF model derives from the fact that the nonlinearity $\sigma$ is a function of *x*, because it depends on the local average $g*I(x)$. As a consequence, the INRF model can **not** be expressed as a weighted average of the input passed through some nonlinearity: the same point $y_i$ will have a different nonlinear transform applied when it is contributing to point $x_1$ than when it is contributing to point $x_2$, i.e. $\sigma (I(y_i)-g * I(x_1))$ vs $\sigma (I(y_i)-g * I(x_2))$. This also implies that the INRF model is **not** shift invariant, and therefore it can **not** be expressed as a convolution. In other words, the INRF can’t be expressed as a usual two-layer neural network, and for this reason we say that the INRF is *intrinsically* nonlinear and fundamentally different from the traditional nonlinear subunit summation models pioneered by Shapley and Victor^40^, as those models do follow the regular L+NL paradigm.

In fact, it would be extremely inefficient to express the INRF in the conventional L+NL framework. To understand the problem, let us concentrate on the term of INRF with the nonlinearities, call it *R*(*x*), and assume for simplicity that the kernel *g* is very narrow and can be considered like a delta function:

4 $$\begin{aligned} R(x) = \sum _i w_i \sigma (I(y_i) - I(x) ) \end{aligned}$$

In order to compute the response at location *x*, *R*(*x*) adds up the contributions from all neighbors $y_i$. For each $y_i$, *R*(*x*) computes the difference between $I(y_i)$ and *I*(*x*), passes this difference through the nonlinearity $\sigma$, and multiplies this by the weight $w_i$ that depends on *x* and $y_i$: the resulting number is what $y_i$ contributes to *R*(*x*). If we wanted to express *R*(*x*) using filters, we would need to have a different filter for each neighbor $y_i$, with each of these filters $F_i$ being zero everywhere except for having a value of $+1$ at $y_i$ and a value of $-1$ at *x*:

5 $$\begin{aligned} I(y_i) - I(x) = F_i * I \end{aligned}$$

Next the nonlinearity $\sigma$ would have to be applied to the result of each filtering operation, yielding an intermediate image *J* where the value at each location $y_i$ is

6 $$\begin{aligned} J(y_i)=\sigma (F_i*I) \end{aligned}$$

Finally, this image should be convolved with kernel *w*, producing the response at each location *x*:

7 $$\begin{aligned} R(x) = (w * J)(x) \end{aligned}$$

As a result, computing the INRF *for a single point* *x* in an image with *N* discrete locations (or pixels) would require $N+2$ convolutions (one convolution per neighbor plus a final convolution with *w* for *R*(*x*), plus another convolution with *m*), when the classical linear RF needs just one convolution for the whole image, of course. For example, a INRF in a one-megapixel image would require over *a million* linear filters to be expressed in L+NL form.

### A linear RF is a specific case of the INRF

In common scenarios the INRF simplifies to a linear RF. For instance, if $\sigma$ is just a linear scaling ($\sigma (z)=\alpha z$), or when the contrast differences are not too large so that they fall inside the linear range of $\sigma$, then we can show that the INRF model becomes the classical linear RF of Eq. (1):

8 $$\begin{aligned} INRF(x) =&\sum _i m_i I(y_i) -\lambda \sum _i w_i \alpha \left( I(y_i) - \sum _j g_j I(y_j) \right) = \sum _i m_i I(y_i) - \sum _i \lambda \alpha w_i I(y_i) + \sum _j \left( \lambda \sum _i w_i \alpha \right) g_j I(y_j) \nonumber \\ =&\sum _i m_i I(y_i) - \sum _i \hat{w_i} I(y_i) + \sum _j \hat{g_j} I(y_j) = \sum _i (m_i - \hat{w_i} + \hat{g_i}) I(y_i) = \sum _i k_i I(y_i), \end{aligned}$$

where $\hat{w_i},\hat{g_i}$ are just scaled versions of $w_i,g_i$. Furthermore, if the kernels *m*, *g* are Gaussians, and kernel *w* is a sum of a wide and a narrow Gaussian as argued in^41^ for lateral inhibition in the retina, then we see that the resulting kernel *k* can have the form of a Difference of Gaussians (DoG) linear RF, a stalwart in vision science^42^.

### The INRF embodies the efficient representation principle

Sapiro and Caselles^43^ showed that the following partial differential equation (PDE) performs histogram equalization:

9 $$\begin{aligned} I_t(x,t)=\frac{1}{2} - I(x,t) -\lambda \sum _i sign(I(y_i,t) - I(x,t) ), \end{aligned}$$

where *x* denotes the spatial location, the input signal *I* is in the range [0, 1] and $\frac{1}{2}$ is the spatial average of *I*. The steady state of Eq. (9) is an image with a flat histogram:

10 $$\begin{aligned} I(x)=\frac{1}{2} -\lambda \sum _i sign(I(y_i) - I(x) ) \end{aligned}$$

We can see how if we replace the global mean average $\frac{1}{2}$ with a local mean average $\sum _i m_i I(y_i)$, we introduce locality also in the sign summation term through weights $w_i$, we regularize the sign function turning it into a sigmoid $\sigma$, and we consider a very narrow kernel *g* (akin to a delta function), then Eq. (10) turns into Eq. (3) and we have exactly our INRF model.

Thus, we can argue that the INRF approximates local histogram equalization and for this reason it’s an embodiment of the efficient representation principle. Therefore, modeling the RF of retinal ganglion cells with a INRF could explain how these neurons are able to perform a constrained form of histogram equalization in the very short time interval between rapid eye movements^8^, at the same time providing another justification for the center-surround properties of their RFs^44^ when the kernels *m*, *w*, *g* are Gaussians. Histogram equalization achieves a similar statistical goal as divisive normalization, a canonical computation in the brain^26^ that sets the neural responses to more finely cover the available range and to become invariant to global properties such as lightness, contrast or orientation texture^30^. But histogram equalization (of the distribution of the norm of neural signals) is better at reducing redundancy than normalization, and the INRF is biologically plausible unlike other mechanisms for redundancy reduction that are also derived from Barlow’s principle such as radial factorization^45^. In image processing, histogram equalization is a classical technique to improve image appearance and it has been used in a variational model for perceptual color correction^46^, whose gradient descent equation has a term that is a particular instance of the INRF model; this links as well our INRF formulation with the very effective color enhancement model of Rizzi et al.^47^.

Furthermore, we can relate the INRF to another successful vision science theory, that of the Wilson–Cowan equations^48^, which have a long and thriving history of modelling cortical low-level dynamics^28^. It has recently been shown^49^ that the Wilson–Cowan equations are not variational, in the sense that they can’t be minimizing an energy functional. The simplest modification that makes them variational yields a model whose main term has the form of an INRF. This modified model accomplishes local histogram equalization, and predicts brightness perception phenomena better than the original Wilson–Cowan model.

### Applications of the INRF for vision science

The INRF has a key property of wide-ranging implications, our results show: for several vision science phenomena where a linear RF must vary with the input in order to predict responses, the INRF can remain constant under different stimuli.

#### Visual neuroscience

A constant INRF can qualitatively explain the very puzzling neurophysiological data by which OFF cells in V1 turn into ON cells when the spatial frequency of the stimulus increases^20^.

We recall that neurons in the ON pathway respond to stimuli above the average level, while neurons in the OFF pathway respond to stimuli below the average level. Under the standard model, the experimental data in^20^ is quite striking because it suggests that the neuron changes the pathway it belongs to (ON or OFF) depending on the input, and modeling this phenomenon with a linear RF requires not just a minor modification of the RF with the input but a complete inversion of polarity of the RF as the spatial frequency changes.

**Figure 2:**
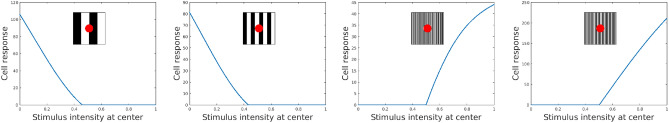
OFF cells become ON cells when the spatial frequency of the stimulus increases. Each panel plots the cell response at the center region as a function of the input value at said region, stimulus shown in inset (red circle denotes center region, which has a gray value inside the input range). The response is computed in this manner: the input stimulus is convolved with a small Gaussian to simulate retinal blur, then the INRF is applied to it and finally the result is rectified (see “Methods”). Notice how in the left and middle-left panels the cell behaves as an OFF cell, since it responds only to stimuli below the average level of 0.5, while the reverse happens for the middle-right and right panels, where the cell responds only to stimuli above the average and therefore behaves as an ON cell.

With the INRF formulation, on the other hand, we can use a fixed INRF and predict the data: when the stimulus spatial frequency is low the INRF responds to inputs below the average, and when the spatial frequency is high *the same* INRF responds to stimuli above the average; see Fig. 2. In this case the nonlinearity $\sigma$ in our model is approximately a sinusoid (see “Methods”), hinting at possible connections of the INRF with the nonlinear time series analysis of oscillations in brain activity^50^.

#### Visual perception

**Figure 3:**
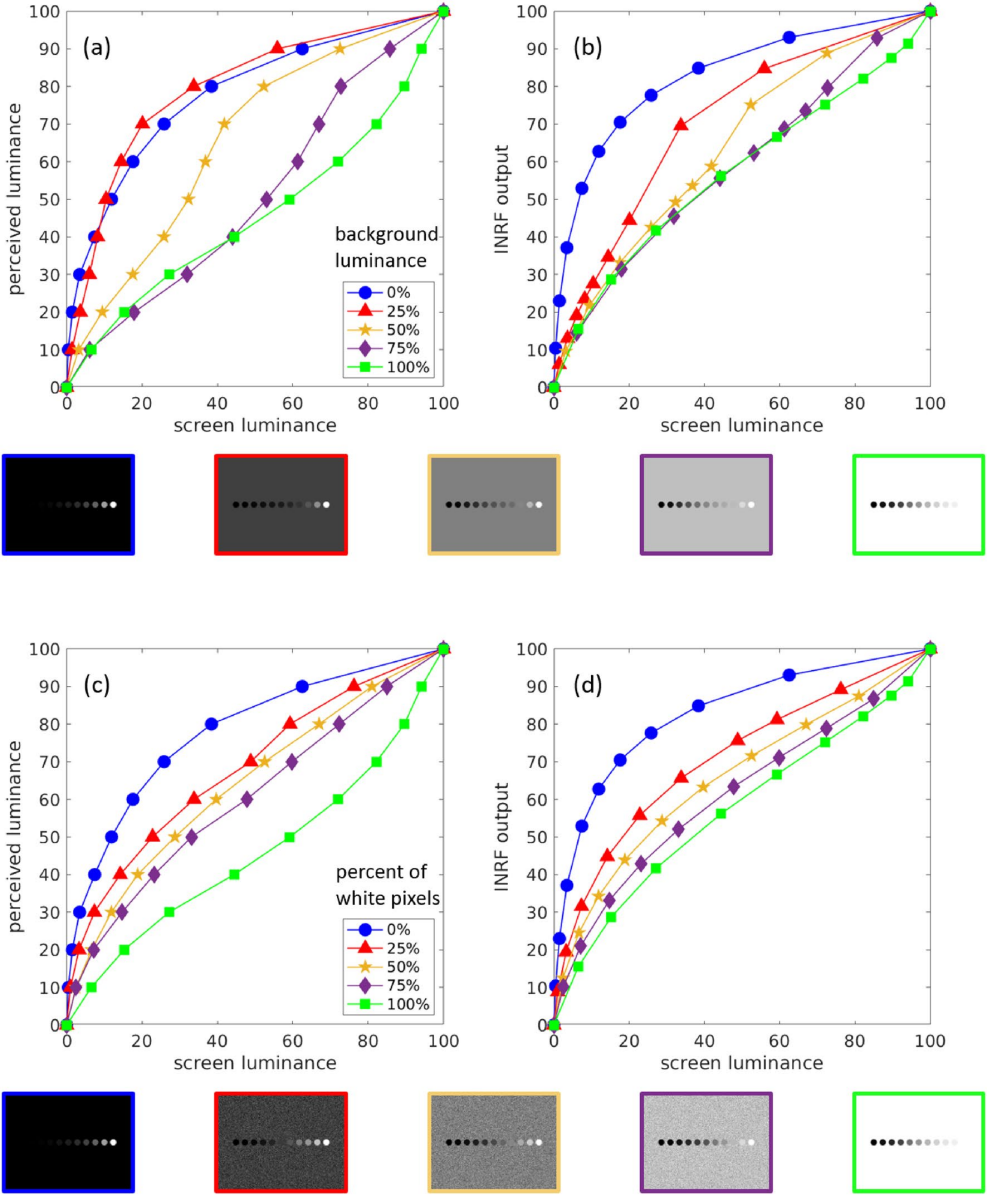
Brightness perception curves (average over all observers) show “crispening” at the surround luminance level when the background is uniform (**a**), but the phenomenon disappears for salt and pepper background (**c**). A model based on the INRF that uses Gaussian kernels for *m* and *w* qualitatively replicates both cases (**b**,**d**) with a fixed set of parameters, which is not possible with a DoG, linear RF formulation.

**Figure 4:**
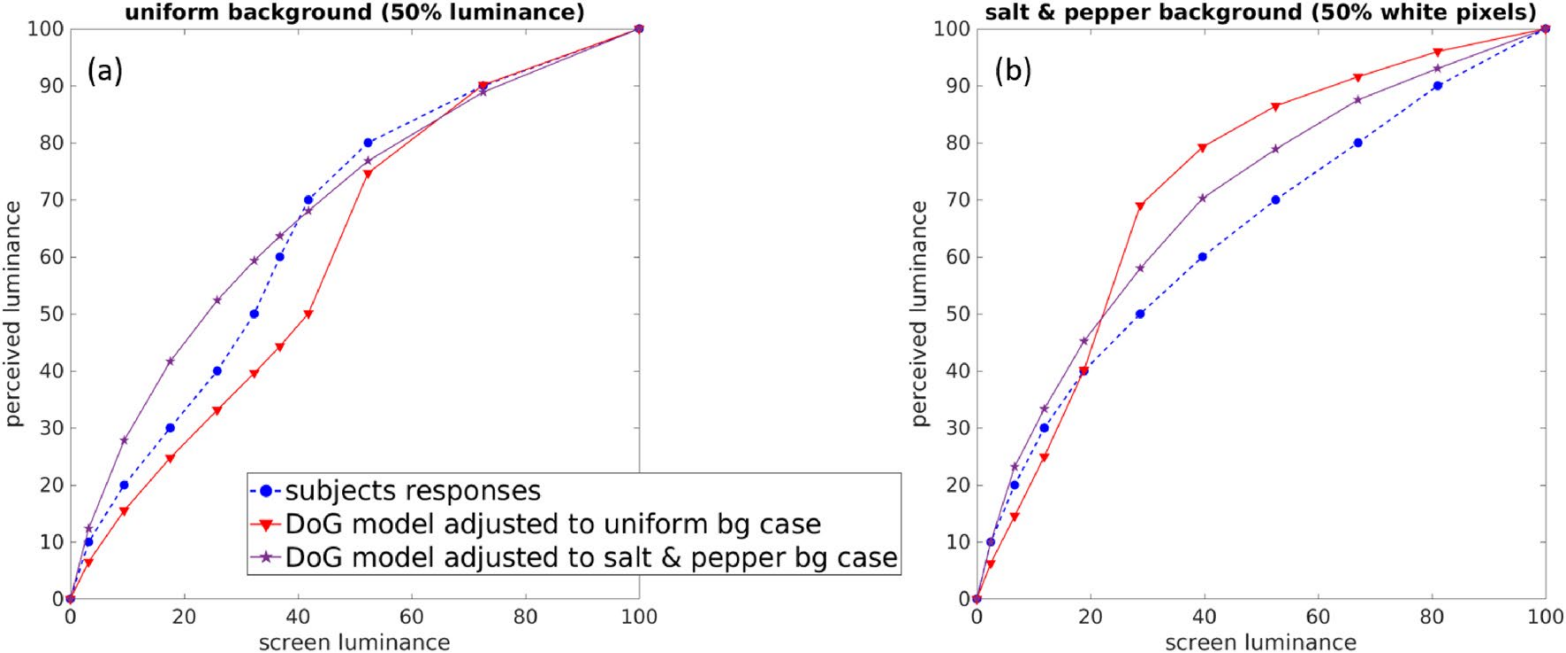
A simple L+NL model, consisting of a difference of Gaussians (DoG) linear RF followed by a pointwise nonlinearity, has to change with the input in order to reproduce the crispening phenomenon. When the model is adjusted to the uniform background condition (red curve), it qualitatively replicates brightness perception for the uniform background case (blue curve, left) but not for the salt and pepper surround case (blue curve, right). The reverse happens when the model is adjusted for the salt and pepper background condition. Both the DoG filter and the nonlinearity change with the stimuli (see “Methods”).

Figure 3a shows the result of a psychophysical experiment. Participants were asked to adjust the luminance values of a series of circular patches lying over a uniform surround (example images are shown in the second row of the figure) until all brightness steps from one circle to the next are perceived to be identical from black to white, i.e. observers create a uniform brightness scale. Each curve in Figure 3a represents, for a given uniform background, the average over all observers of the brightness perception function. We can see how the slope of each brightness perception curve increases around its corresponding background luminance level. This effect is called “crispening”, and it’s a very complicated perceptual phenomenon to model^51^ as it’s very much dependent on the input. For instance, if in the experiment above the uniform surround is replaced by salt and pepper noise of the same average (example images are shown in the bottom row of the figure), the crispening virtually disappears, see Fig. 3c.

We have found that the same INRF, i.e. using a fixed set of parameters for our model, can adequately predict how crispening happens with uniform backgrounds, see Fig. 3b, and how it is abolished when the background is salt and pepper noise, see Fig. 3d. Our extremely simple brightness perception model consists of just two stages: the first one is a Naka–Rushton equation to model photoreceptor response^42^, and the second step is a INRF that models the responses of retinal ganglion cells, where kernels *m*, *w* are simple Gaussians, *g* is a Dirac delta and the nonlinearity $\sigma$ is an asymmetric sigmoidal function with different exponents for the positive and negative regions (see “Methods”).

If, after the Naka–Rushton stage, one were to use the classical L+NL formulation with a Difference of Gaussians (DoG) kernel and a pointwise nonlinearity instead of the INRF, it would be possible to optimize its parameters so that the L+NL model fits the psychophysical data and predicts crispening for the uniform background condition, as shown by the red curve in Fig. 4a. But then this L+NL model would not be able to replicate the psychophysical data for the salt and pepper surround, as seen in Fig. 4b: it still predicts crispening in this case, when for observers crispening has disappeared. Conversely, we can fit the L+NL model to the salt and pepper background condition, obtaining a different set of parameters than for the uniform background condition (see “Methods”). Now, as expected, the L+NL model qualitatively replicates the perceptual data for the salt and pepper surround (magenta curve in Fig. 4b) but not for the uniform background case (see Fig. 4a).

We can use the INRF-based model for brightness perception just described, call it INRF-B, to produce an image quality metric, call it INRF-IQ, in the following way. Given a reference image *I* and a distorted version of it $I_D$ (e.g. $I_D$ may have noise, blur, compression artifacts, etc.), we pass each of them through INRF-B and then compute the root mean square error (RMSE) between the results: INRF-IQ($I,I_D$) = RMSE(INRF-B(*I*),INRF-B($I_D$)). Despite the fact that INRF-B has only five parameters (namely $\lambda$, the standard deviations of the Gaussians *m* and *w* and the two exponents of $\sigma$) that have been optimized for the brightness perception experiment in less than 10 synthetic images, INRF-IQ reaches a correlation value with the mean opinion scores (MOS) of observers on the large scale database TID2013^52^ that is comparable to the correlation of a state-of-the-art deep learning perceptual metric like LPIPS^53,54^, that has 24.7 million parameters and has been trained on more than 160, 000 natural images, with close to 500*K* human judgements for labeling pair-comparison preferences. See Table 1 and “Methods”. With a further refinement of the five parameters w.r.t. the TID2013 database, the INRF-IQ achieves a $77\%$ correlation with the MOS. Very importantly, when the INRF-IQ is implemented as a sequential stack of two INRF-B modules (i.e. having 10 parameters) the correlation with the MOS increases to $81\%$, showing that the addition of concatenated layers is beneficial for the INRF formulation, like it is for linear RFs.

**Table 1:** Pearson correlation with Mean Opinion Scores (MOS) in TID2013 database^52^ for different image quality metrics: PSNR, SSIM^55^, LPIPS^53,54^, INRF-IQ (proposed in text).

	PSNR	SSIM	LPIPS	INRF-IQ
Correlation with MOS	57%	65%	76%	74%

**Figure 5:**
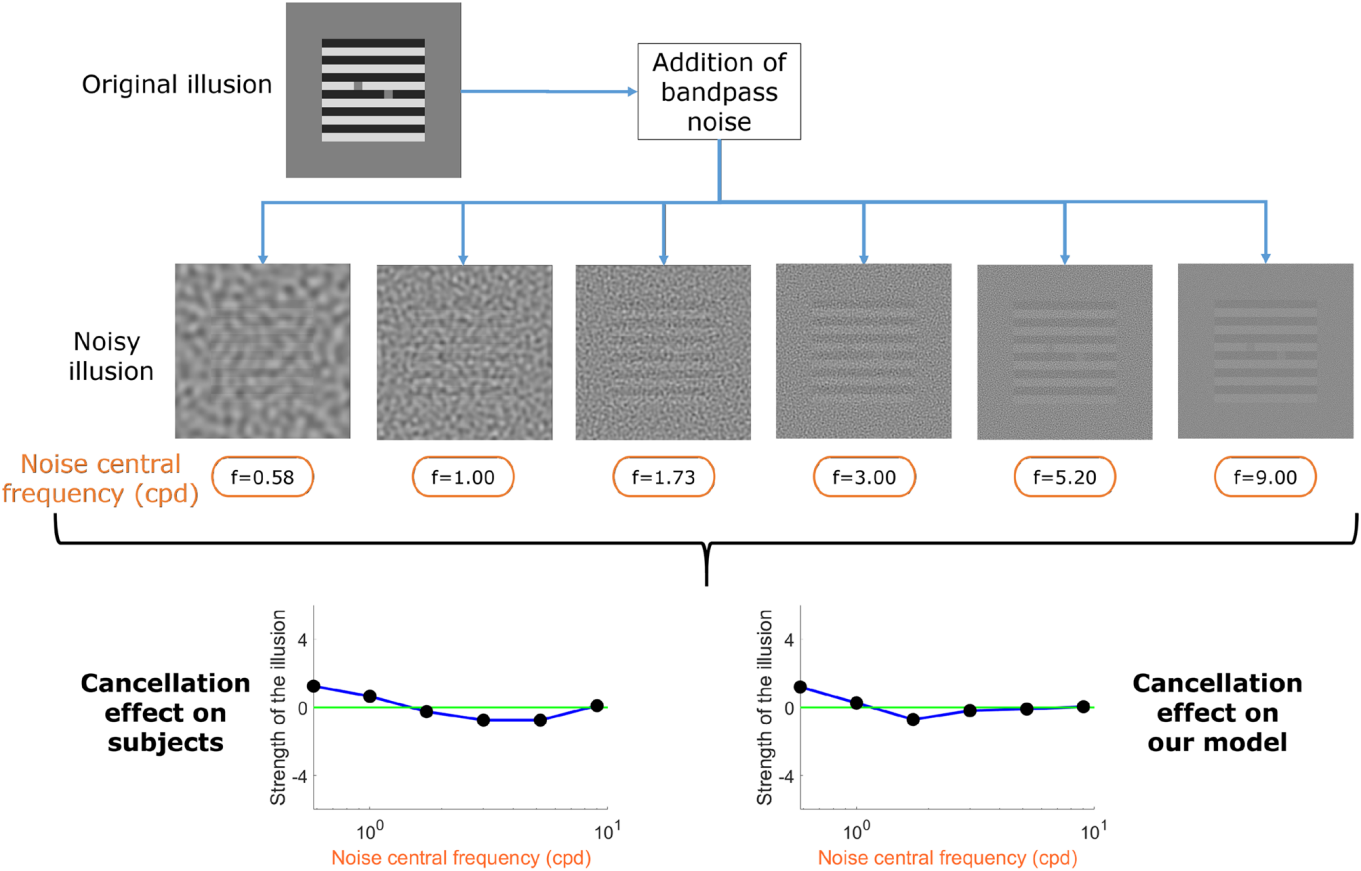
A model based on the INRF qualitatively predicts the observers’ response to White’s illusion when bandpass noise is added. Given 6 different levels of bandpass noise frequency, our model presents the same trend as the observers’ data published in^56^. This is particularly striking when comparing our results with those shown in^56^, where none of the vision models that were tried, based on linear RFs, were able to replicate this behaviour.

Another very interesting example is that of White’s illusion, a brightness illusion in which two identical gray areas are perceived as being different due to their disparate surrounds. Vision models based on a linear RF can reproduce the illusion, but they fail to match the psychophysical data when bandpass noise is added to the image^56^. The INRF model with a fixed set of parameters is able to replicate White’s illusion and predict the observers’ response when different types of bandpass noise are added, see Fig. 5 (the INRF also allows to predict this data if incorporated into a neural field model as done in^49^).

**Figure 6:**
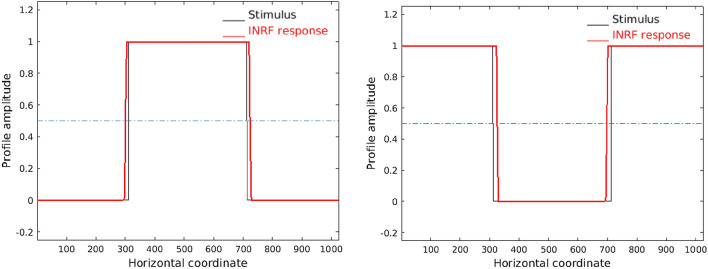
Light/dark asymmetry (*“irradiation illusion”*): a white square over black background (left) is perceived as being larger than a black square over white background (right). This phenomenon can be reproduced with a L+NL model that changes with the stimulus^57^. Instead we model the irradiation illusion with a fixed INRF followed by clipping in the range [0, 1] (see “Methods”).

We have been able to qualitatively reproduce as well the perceived spatial asymmetries for lights and darks using a constant INRF. In the *“irradiation illusion”*, a white square over black background is perceived as being larger than a black square over white background. This phenomenon can be reproduced with a L+NL model that changes with the stimulus^57^, but we are able to model this illusion simply with a fixed INRF, see Fig. 6.

Remarkably, the results shown in Table 1, Figs. 3, 5 and 6 have all been obtained with the same set of parameter values (see Table 5): a standard deviation of $0.81^\circ$ for *m*, a standard deviation of $2.77^\circ$ for *w*, a value of $\lambda =3.875$, *g* being a Dirac delta and the nonlinearity $\sigma$ being $\sigma (z)=z^{0.625}$ when $z\ge 0$ and $\sigma (z)=-|z|^{0.775}$ when $z<0$ (for details on the optimization process to find these parameter values see “Methods”).

All the visual perception experiments considered are instances of brightness perception, and the INRF units used could be thought as modeling retinal ganglion cells. Hence they are preceded by some sort of photoreceptor nonlinearity, but that is not a central part of the model, it is only required in this brightness context. An indication that this is not a crucial part of our model lies in the fact that different photoreceptor nonlinearities have been used for the four visual perception experiments (see “Methods”), yielding good results with the same INRF applied afterwards. In any case we see INRF as a summation model that could be applied at other layers downstream without this previous nonlinearity.

### Application of the INRF to artificial neural networks

Finally, we have tested the idea of modifying a convolutional neural network (CNN), replacing each of its linear filters by a INRF while keeping all other elements of the architecture the same and maintaining the number of free parameters, then training this new network and comparing the performance with the original CNN. We’ve done this experiment for an image classification task, using two architectures and the four benchmark databases that are regularly used in the literature. As shown in Table 2, in all cases the INRF-based ANN outperforms the CNN in terms of classification error, with wide improvements that go from $10\%$ for MNIST to a remarkable $45\%$ for SVHN. Preliminary tests on a 20-layer residual network using the CIFAR10 dataset show a $5\%$ improvement for the INRF network over the CNN. We have also subjected our INRF-based ANN to four different forms of adversarial attacks, and in all cases it’s remarkably more robust than the CNN, as shown in Tables 3 and 4.

**Table 2:** Comparison of classification error between a CNN and the equivalent network using INRF elements instead of linear RF filters.

Dataset	CNN	INRFnet
MNIST	0.48	0.43
CIFAR10	24.28	16.78
CIFAR100	57.01	48.8
SVHN	6.26	3.41

**Table 3:** Accuracy against whitebox adversarial attacks on the MNIST dataset.

Attack methods	FGSM ($\epsilon =0.1$)	FGSM ($\epsilon =0.2$)	FGSM ($\epsilon =0.3$)	DeepFool	Carlini–Wagner ($L_2$)	Carlini–Wagner ($L_\infty$)
CNN	88.14%	44.69%	11.03%	52.01%	4.18%	42.5%
INRFnet	93.14%	62.23%	33.42%	65.27%	7.24%	58.06%

**Table 4:** Accuracy against whitebox adversarial attacks on the CIFAR10 dataset.

Attack methods	FGSM ($\epsilon =0.05$)	FGSM ($\epsilon =0.1$)	FGSM ($\epsilon =0.15$)	DeepFool
CNN	13.27%	12.26%	10.79%	47.63%
INRFnet	19.3%	16.6%	15.6%	57.46%

## Discussion

We have presented the INRF and demonstrated that it has a number of remarkable advantages with respect to the classical linear RF. The INRF, introduced as a physiologically-plausible single-neuron summation model that is consistent with more recent studies on dendritic computations, embodies the efficient representation principle and provides an explanation for the ability of retinal cells to perform very rapid histogram equalization. For several phenomena in visual neuroscience and visual perception, the INRF is shown to have the capability of remaining constant for different inputs while the linear RF must change with the stimulus, indicating that the INRF might actually be determined by the cell’s wiring. Furthermore, we have been able to find a set of parameter values with which a single INRF element (emulating the output of retinal ganglion cells) is able to qualitatively predict perceptual responses in situations as diverse as the “crispening” effect, appearance of visual distortions in an image quality database, White’s illusion and the irradiation illusion. This supports the argument that the INRF is an adequate form of RF to model visual phenomena, and suggests that, as with the linear RF, better predictions could be achieved by stacking INRF elements in cascade, so that results in one stage are refined in the next. The INRF is also shown to be extremely effective in improving the performance of ANNs in terms of classification error and robustness to adversarial attacks.

In many common scenarios the INRF reduces to the linear RF, but in general it can’t be expressed efficiently in L+NL form. In our opinion, this has been the key reason why a model like INRF has not been proposed before, despite the wide variety of approaches that over the years have been introduced to model the nonlinear nature of visual phenomena: as all these vision models follow the L+NL paradigm (because they are grounded in the notion of a linear RF), they are not able to implement operations like the ones involved by the INRF unless there is an explosion in the dimension of the linear part. Furthermore, we argue that a model like INRF would most definitely not have appeared if we had followed the current trend of studying vision by using deep ANNs^37^: while it is true that goal-driven ANNs are the state of the art in predicting cortical responses, by their own design they can’t yield a model that falls outside the regular L+NL paradigm. While possible in theory, in practice the chances of obtaining the INRF from the hidden layers of an ANN trained for some visual task are virtually zero, as this could only come after designing and training a colossal, unprecedented in size and highly inefficient network architecture.

There are many interesting questions that spring up from our proposed model, some of which we’re currently investigating. We’re also interested in analyzing how changes in $\sigma$ affect the model, and specifically if there are any advantages in a generalized version of it where the nonlinearity is not just shifted but also allowed to change in shape as a function of spatial location *x*. For visual perception, we are assessing the ability of our model to predict color appearance phenomena. Another important aspect is that of transmission of information, which goes beyond redundancy reduction (or histogram equalization) in the response^58^. Future research should quantify the ability of INRF layers in information transmission from the stimuli into the internal image representation, where the nature of the noise in INRF units (not considered in this work) will be of paramount relevance, following^59,60^. And for ANNs with INRF modules, we’re studying other configurations (GAN, autoencoder and a recurrent version), the mathematical properties of the INRFnet that make it more resistant to adversarial attacks, the potential of an INRFnet to replicate visual illusions better than CNNs^61^, and INRFnet implementations for input signals other than images.

To conclude, our results suggest a change of paradigm for vision science as well as for artificial intelligence, and open up groundbreaking opportunities for vision research that can potentially bring a game-change in the field, by constituting the new building block with which to produce neural models that perform better in predicting the responses to natural stimuli. The potential effect of fully reaching this goal is immense for vision science, allowing to better understand how the brain processes signals, to help establish and analyze neural circuits, to see in which way neural signals relate to visual perception, and to develop accurate models of image appearance. This in turn would permit to create methods to produce uniform test batteries for easier replication of experiments in neuroimaging and psychophysics, develop better image processing and computer vision algorithms for many different key problems simply by minimizing perceptual error metrics, manufacture cameras and displays that faithfully match our perception, tailor them to individuals suffering from a visual deficiency, etc. The INRF can allow to create ANNs that perform more similarly to a human observer, in the sense that they become robust to adversarial attacks, easier to train, and have better generalization properties. These are all some of the most significant problems currently affecting ANNs, hence the potential impact of our proposal may be quite substantial given the ubiquity of ANNs in research and the industry.

## Methods

**Table 5:** Summary of the vision science experiments performed with different instances of the INRF model and the corresponding choice of parameters.

Experiment	Nonlinearity on input	Kernel *m*	Kernel *w*	Kernel *g*	$\lambda$	Nonlinearity $\sigma (\cdot )$
OFF/ON cells	None	Constant (size = 85 px)	Constant (size = 512 px)	Constant (size = 85 px)	1000	$sin(\pi z)$ if $|z|<0.5$, $sign(z)*(sin(\pi z))^2$ otherwise
Crispening	Naka–Rushton eq. (semisaturation from^62^, n = 0.74)	Gaussian (std = $0.81^\circ$)	Gaussian (std = $2.77^\circ$)	Delta function	3.88	$z^{0.63}$ if $z\ge 0$, $-|z|^{0.78}$ if $z<0$
Image quality metric	Power law (lightness channel from CIELAB)	Gaussian (std = $0.81^\circ$)	Gaussian (std = $2.77^\circ$)	Delta function	3.88	$z^{0.63}$ if $z\ge 0$, $-|z|^{0.78}$ if $z<0$
White’s illusion	Power law (gamma-corrected data)	Gaussian (std = $0.81^\circ$)	Gaussian (std = $2.77^\circ$)	Delta function	3.88	$z^{0.63}$ if $z\ge 0$, $-|z|^{0.78}$ if $z<0$
Irradation illusion	Naka–Rushton eq. (semisaturation = 18, n = 1)	Gaussian (std = $0.81^\circ$)	Gaussian (std = $2.77^\circ$)	Delta function	3.88	$z^{0.63}$ if $z\ge 0$, $-|z|^{0.78}$ if $z<0$

Except for the OFF/ON cells experiment (first row), the results for the other four experiments have all been obtained with the same set of parameter values.

### OFF cells become ON cells when the spatial frequency of the stimulus increases

The plots in Fig. 2 were obtained in the following manner. We consider a 1D stimulus *I* of width 512 points, consisting of alternating black and white segments of width **b** as background and a superimposed center segment of value **u** and width 64. In order to simulate retinal blurring, *I* is convolved with a Gaussian kernel of standard deviation 4, yielding $I_b$. Next, the INRF model is applied to $I_b$ with the following parameters: the kernels *m* and *g* have a width of 85 and a constant value of 1/85, the kernel *w* has a width of 512 and a constant value of 1/512, $\lambda =1000$ and the nonlinearity $\sigma$ has the form $\sigma (z)=sin(\pi z)$ if $|z|<0.5$, otherwise $\sigma (z)=sign(z)*(sin(\pi z))^2$. This result is rectified, yielding the final output *O*, our model of the cell response, whose center point has some value **v**. We repeat this process for different values of the center region in the stimulus, varying **u** from 0 (black) to 1 (white). Each panel in Fig. 2 plots the resulting curve of cell response values **v** as a function of stimulus intensity **u**, for a given bar width **b**.

### Modeling crispening with the INRF

#### Apparatus and subjects

Stimuli were displayed on a Philips 109B CRT monitor of 1280 by 960 pixels and 75 Hz and a luminance range from 0.65 to 75 cdm$^{-2}$ in a purpose built laboratory. The display was viewed at a distance of 58 cm so that 64 pixels subtended 1 degree of visual angle. There were 13 participants in total, all with corrected to or normal vision. Informed consent was obtained from all participants. All procedures complied with the declaration of Helsinki and were approved by the ethical committee of the host institution: Comité Ético de Investigación Clínica, Parc de Salut MAR, Barcelona, Spain.

#### Psychophysical experiments on perceptual linearisation

Subjects viewed eleven uniform circles with 1$^{\circ }$ diameter laying upon the horizontal meridian of the monitor and separated by 1$^{\circ }$ horizontal gaps. The leftmost circles were always black and the rightmost circles always white and the intermediary circles began with a random luminance value. There were two psychophysical experiments performed. The uniform background luminance levels in Experiment 1 were 0.65, 19.24, 37.83, 56.41, 75.00 cd/m$^{2}$. Experiment 2 used a salt (white) and pepper (black) background and the ratio of black to white pixels was varied from 0 to 100%. Each subject had to manipulate the luminance of the nine intermediary circles until they appeared to vary, from left to right, in a perceptually linear manner from black to white.

#### Replicating the psychophysical data using an INRF model

The brightness perception curves produced by the INRF model in Fig. 3 were obtained as follows. Let $L_k$ represent one of the stimuli images shown in the second and fourth rows of the figure, taking values in the range [0, 100]. We first apply a Naka–Rushton equation modelling photoreceptor response^42^: $I_k(x) =\displaystyle {L_k(x)^n}/{(L_k(x)^n+S^n)}$, where $n = 0.74$ and the semi-saturation constant *S* is calculated using the formula $S = 0.5\cdot 18 + 0.5\cdot 10^{0.63*log10(b_k)+1}$ obtained from^62^, where $b_k$ is the average luminance value of the input $L_k$. Then, the INRF model from Equation (3) is applied to $I=I_k$, yielding an output image $O_k$. The parameters for the INRF model are the following: *m* and *w* are Gaussian kernels with standard deviation equal to 52 and 178 pixels, respectively, *g* is a delta function, $\lambda = 3.875$, and the nonlinearity $\sigma$ is $\sigma (z)=z^{0.625}$ when $z\ge 0$ and $\sigma (z)=-|z|^{0.775}$ when $z<0$. Each curve in Fig. 3b,d plots the value of a $O_k$ image at the center of the eleven circles.

Results shown in Fig. 4 are obtained by adjusting a simple L+NL model:

11 $$\begin{aligned} LN(x)=\sum _i m_i I(y_i) -\lambda \sigma ( \sum _j DoG(y_j-x) I(y_j)), \end{aligned}$$

with *m* a linear kernel, *DoG* a kernel defined as a difference of two Gaussian kernels, $\sigma$ a sigmoid function and $\lambda$ a scalar parameter balancing both terms. The L+NL model of Eq. (11) is applied to the output $I_k$ of the Naka–Rushton equation with the same parameters as mentioned above. For the uniform background case (Fig. 4a), *m* is a Gaussian kernel with standard deviation equal to 52, the Gaussian kernels that compose *DoG* have a standard deviation of 30 and 60 pixels respectively, $\lambda$ is equal to 2 and $\sigma$ is $\sigma (z)=z^{0.5}$ when $z\ge 0$ and $\sigma (z)=-|z|^{0.7}$ when $z<0$. In the case of salt and pepper background, *m* is again a Gaussian kernel with standard deviation equal to 52, the standard deviation of the Gaussian kernels of *DoG* is 40 and 50 pixels respectively, $\lambda =10$ and $\sigma$ is $\sigma (z)=z^{0.9}$ when $z\ge 0$ and $\sigma (z)=-|z|^{0.9}$ when $z<0$. The plotted curves represent the output value of the model at the center of each of the eleven circles.

### A simple image quality metric based on the INRF

The results reported in Table 1 were obtained as follows.The images from the TID2013 dataset and the values for MOS, PSNR and SSIM for each image were downloaded from the webpage of the authors of^52^. Pearson correlation with MOS in TID2013 for LPIPS was obtained from^54^. The INRF-IQ metric for a pair of reference *R* and distorted *D* images is calculated as follows. The images are converted from sRGB to CIELAB color space. Note that transform to CIELAB to compute lightness plays the role of the Naka–Rushton photoreceptor nonlinearity used in the other experiments. Their respective lightness channels ($L_R$ and $L_D$) are then passed through the INRF model (3) with the same parameters of the brightness perception experiment, i.e. *m* and *w* are Gaussian kernels with standard deviation equal to 52 and 178 pixels, respectively, *g* is a delta function, $\lambda = 3.875$ and the nonlinearity $\sigma$ is $\sigma (z)=z^{0.625}$ when $z\ge 0$ and $\sigma (z)=-|z|^{0.775}$ when $z<0$. The application of the INRF model to $L_R$ and $L_D$ yields, respectively, the output images $O_R$ and $O_D$, and the root mean square error between them is our proposed image quality metric: $\text {INRF-IQ}(R, D) = \text {RMSE}(O_R, O_D)$.

### Modeling White’s illusion with noise using the INRF

The results for this experiment are shown in Fig. 5. They were obtained in the following manner. The goal was to model the results presented in^56^ where 6 different levels of bandpass noise were added to the original illusion. We follow the same paradigm proposed in there for showing the strength of the illusion in vision models. In particular, for each noise level, they compute the result of the different methods for 25 realizations of the noisy image and average them in order to guarantee the results are stable. From this average image, they compute the mean in the two grey-level patches where the illusion occurs. Finally, the strength of the illusion is defined as the difference between these two values, but with a fixed polarity that is defined as the polarity found in the original White illusion. In our case, we use the INRF model explained for crispening (but without the Naka–Rushton part, as we are emulating the experiment of^56^ in which there is not need to convert to photoreceptor values), and we also compute our model on 25 noisy realizations in order to have stable results. As seen in Fig. 5 our model presents the same trend as the observers’ data. This is particularly striking when comparing our results with those shown in^56^, where none of the state-of-the-art linear RF vision models that were tried were able to replicate this behaviour with fixed parameters. Our parameters are the same -in terms of visual angles- as in the crispening and image quality tests, and they are the following: kernel *m* is a Gaussian of standard deviation 54 pixels, kernel *w* is a Gaussian of standard deviation 183 pixels, *g* is a Dirac delta, the nonlinearity is $\sigma (z)=z^{0.625}$ when $z\ge 0$ and $\sigma (z)=-|z|^{0.775}$ when $z<0$, and $\lambda =3.875$.

### Modeling the irradiation illusion with the INRF

The results in Fig. 6 were obtained in the following manner. This is a 1D example where the input stimulus has width 1024 points, values 0 or 1, with a central step (corresponding to the square in the 2D case) of width 400 points. First, the input is convolved with a Gaussian of standard deviation 20 to emulate retinal blur. Then, the photoreceptor response is emulated with a Naka–Rushton equation of the form $NR(v)=v/(v+0.18)$, where the semi-saturation constant has been chosen to be 0.18 as it corresponds to a mid-gray value. Next, the INRF is applied with the following parameters: kernel *m* is a Gaussian of standard deviation 100, kernel *w* is a Gaussian of standard deviation 342, *g* is a Dirac delta, the nonlinearity is $\sigma (z)=z^{0.625}$ when $z\ge 0$ and $\sigma (z)=-|z|^{0.775}$ when $z<0$, and $\lambda =3.875$. Finally, the result is clipped within the range [0, 1].

### Optimization process for the INRF for visual perception

The results shown in Table 1, Figs. 3, 5 and 6, correspond to the application of the INRF model to the “crispening” effect, an image quality metric, White’s illusion and the irradiation illusion, respectively. In this instance of the INRF model kernels *m*, *w* are simple Gaussians, *g* is a Dirac delta and the nonlinearity $\sigma$ is an asymmetric sigmoidal function with different exponents *p*, *q* for the positive and negative regions ($\sigma (z)=z^p$ when $z\ge 0$ and $\sigma (z)=-|z|^q$ when $z<0$). All these results have been obtained with the same set of parameter values (see Table 5): a standard deviation of $0.81^\circ$ for *m*, a standard deviation of $2.77^\circ$ for *w*, $\lambda =3.875$, $p=0.625, q=0.775$. The optimization process to find these parameters values was as follows.

First, for the “crispening” effect, the standard deviation of *m* was chosen to match the radius of the circular patches and the standard deviation of *w* was chosen to match the distance between patches so that neighboring patches had no influence; the parameters $\lambda ,p,q$ were selected by visual inspection of the results. This set of parameter values provided also excellent results for the image quality metric application, but were found to be sub-optimal for White’s illusion. Then, the parameters were optimized (using Matlab’s *fmincon* function) so as to provide a good fit of the INRF results to the observer data on White’s illusion. This final set of parameter values, which is the one detailed above, was verified to work also for the “crispening” effect and the image quality metric, as well as for the irradiation illusion.

### An INRF-based convolutional neural network

The INRF model can be fully integrated within a convolutional neural network (CNN) framework. To do so, we just replace the convolution with linear filters in the convolutional layers by INRF-modules based on the proposed model (Eq. (3)) and we keep all other elements of the architecture the same (activation functions, batch normalization, dropout, and fully connected layers). Each of these INRF-modules is a multi-channel implementation of Eq. (3) where the kernel *g* is just a Delta function and *m* and *w* are the same kernel, which is the one to be learned. More details on this formulation can be found in the Supplementary Material S1. The code for our implementation can be downloaded from https://github.com/alviur/INRF.

In order to compare the performance of our proposed model with regards to a standard CNN we present a case study on image classification. More in detail, we look at four standard datasets: MNIST^63^, CIFAR10^64^, CIFAR100^64^, and SVHN^65^ without any preprocessing (e.g. whitening or histogram equalization) or data augmentation strategy (input images are normalized in the range $\left[ 0,1\right]$). Both our model and the standard CNN present the same general architecture, as we just replace the convolutions (and bias) by the INRF-modules. The architecture in the case of the MNIST database has 2 convolutional layers followed by a fully-connected layer. The convolutional layers have $5\times 5$ filter size with 32 and 64 channels. After each convolutional layer, $2\times 2$ max pooling and ReLU activation layers are used. Finally a fully-connected layer with 500 hidden nodes is used. In the case of the CIFAR10, CIFAR100, and SVHN datasets, we use a CNN architecture (following^66,67^) with 3 convolutional layers (192 channels in all the layers), followed by a global average pooling with kernel size 8 before a fully-connected output layer. Additionally to activation and pooling layers (disposed after the first two layers), batch normalization layers were added after each convolutional layer.

In MNIST, CIFAR10, and CIFAR100 we use a train-validation split strategy (the training set corresponds to 90% of training data and validation to 10%) to find the best parameters and report performance in the test set. For SVHN we prepared the data as seen in previous works: 400 samples per class from the training set and 200 samples per class from extra set are used as validation set, the remaining 598,388 images (mixing up training and extra set) are used for training. We report the error in the test set using the best model in the validation set. We train in all the datasets using Adam optimizer and a $\lambda$ value (for INRFnets) of 1.1 for MNIST, 2.0 for CIFAR10 and CIFAR100, and 1.0 for SVHN. Table 2 presents the results in terms of the percentage of classification error in each dataset using a CNN and the same architecture with INRF (INRFnet). The INRF always reduces the error of the equivalent CNN architecture, with an improvement that ranges between $10\%$ and $45\%$. Finally, in preliminary tests for the case of a 20-layer CNN, we use the same architecture, hyperparameters, data preprocessing, data augmentation and training procedure used by He et al.^68^ in their ResNet20 CIFAR10 experiment. We use $\lambda =0.1$ and a weight decay of 0.0003 for the INRFnet, and obtain an improvement of $5\%$ over the CNN, the error going down from $9.4\%$ to $8.9\%$.

Results from Tables 3 and 4 were obtained from attacking the CNN and INRFnet that obtained the best accuracy results for the MNIST and CIFAR10 datasets respectively in the previous experiment. We used four white-box adversarial attacks: the Fast Gradient Sign Method (FGSM)^69^, DeepFool^70^, and Carlini & Wagner $L_2$ and $L_\infty$ methods^71^ in the MNIST-trained networks and FGSM and DeepFool for CIFAR10-trained networks. For all the attacks we used their implementations from the Adversarial Robustness Toolbox (ART)^72^.

## Supplementary information

Supplementary material 1
