# Supplementary material for: Evidence for the intrinsically nonlinear nature of receptive fields in vision
Source: Sci Rep. 2020 Oct 1;10:16277. doi: 10.1038/s41598-020-73113-0 (PMC7530701; doi:10.1038/s41598-020-73113-0)
Supplement: Supplementary file 1 — Supplementary material 1 [file 41598_2020_73113_MOESM1_ESM.pdf]

Supplementary material for "Evidence for the intrinsically nonlinear nature of receptive fields in vision"

Marcelo Bertalmío[1,*], Alex Gomez-Villa[1], Adrián Martín[1], Javier Vazquez-Corral[1], David Kane[1], and Jesús Malo[2]

[1]Universitat Pompeu Fabra, Spain
[2]Universitat de Valencia, Spain
[*]marcelo.bertalmio@upf.edu

ABSTRACT

This document describes the architecture of the Intrinsically Nonlinear Receptive Field Networks (INRFnets). In this neural network the main operation is based on the recently proposed intrinsically nonlinear receptive field (INRF) model substituting the convolution operation, keystone of the convolutional neural networks (CNN). In the following, we first describe the implementation of an INRF-module and then we describe the way in which these modules are integrated to build a new type of neural network.
The code for our implementation can be found in `https://github.com/alviur/INRF`

1 From the INRF model to an INRF-module

Let us first define the INRF model for 2D input data u, 2D kernels m, w, g, a scalar parameter λ and a fixed nonlinear activation function σ for a specific location x:

$$\mathrm{INRF}(x;u,m,w,g,\lambda,\sigma)=\sum_{i\in I(x)} m_i u(y_i)-\lambda\sum_{i\in I(x)} w_i\sigma\left(u(y_i)-\left(\sum_{j\in J(x)} g_j u(y_i)\right)\right), \tag{1}$$

where $I(x)$, $J(x)$ denote two sets of indexes corresponding to the neighboring pixels in two 2D windows centered on location x (with a slight abuse of notation, they also refer to the corresponding elements of the 2D kernels m, w and g).

Setting g to be one when $y_i = x$ and zero everywhere else, and letting m and w be the same kernel, the equation above reduces to:

$$\begin{aligned}\mathrm{INRF}(x;u,w,\lambda,\sigma) &= \sum_{i\in I(x)} w_i u(y_i)-\lambda\sum_{i\in I(x)} w_i\sigma\left(u(y_i)-u(x)\right)\\ &= \sum_{i\in I(x)} w_i\left[u(y_i)-\lambda\sigma\left(u(y_i)-u(x)\right)\right],\end{aligned} \tag{2}$$

which is the equation that we implement as the INRF-module.

1.1 Implementation details of an INRF-module

An INRF-module should calculate the expression defined in equation (2) for every possible location x. In order to do these operations efficiently we have designed an implementation based on convolutions that allows us to make use of the fast calculation of these operations in modern machine learning code libraries. Please notice that here we use the term convolution (with the notation '*') to describe the operation of point-wise multiplication followed by a sum of all the resulting products as is typical in the neural network literature.

First, let us modify slightly the notation in equation (2); we write:

$$\mathrm{INRF}(x;u,w,\lambda,\sigma)=\sum_{i\in I(x)} w_i\left[u(y_i,x)-\lambda\sigma\left(u(y_i,x)-u(x)\right)\right], \tag{3}$$

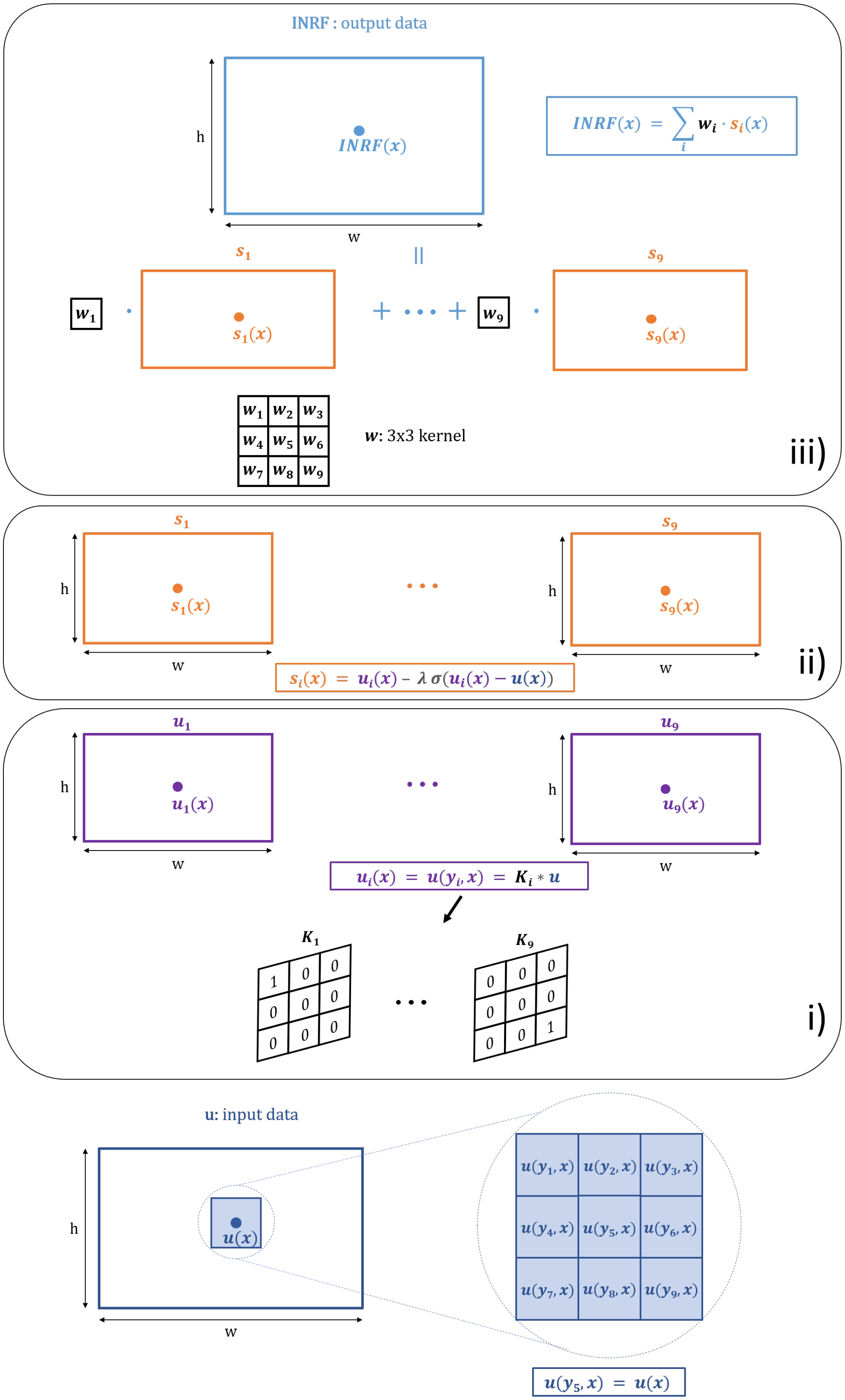

Figure 1. Implementation details of an INRF-module for 2D input and output data.

because the neighbor elements $u(y_i, x)$ are relative to a particular location x. Moreover, we can simplify this notation by defining $u_i(x) = u(y_i, x)$, hence writing:

$$\text{INRF}(x; u, w, \lambda, \sigma) = \sum_{i \in I(x)} w_i \left[u_i(x) - \lambda \sigma \left(u_i(x) - u(x) \right) \right], \tag{4}$$

For the sake of simplicity we will continue this explanation for a 3×3 kernel w, but all these could be generalized for any other kernel size. We also present first the case of having 2D input and output data. We denote as w_i, $i = 1, \ldots, 9$ to the elements of w from top to bottom and left to right, i.e. w_1 is the top left element of the kernel and w_9 the bottom right one. Analogously, $u_1(x) = u(y_1, x)$ is the top left element of the 3×3 window centered in $u(x)$ and $u_9(x) = u(y_9, x)$ is the bottom right element of this same window.

A summary of the implementation is depicted in figure 1. We will unroll the operations from top to bottom. We first define $s_i(x) = u_i(x) - \lambda \sigma (u_i(x) - u(x))$ and then from (4) we have that

$$\text{INRF}(x; u, w, \lambda, \sigma) = \sum_{i \in I(x)} w_i s_i(x), \tag{5}$$

which is **step iii)** in figure 1. Notice that the same value w_i is multiplied by $s_i(x)$ for any position x, so we first calculate the product $w_i \cdot s_i$ and we then sum them pointwise to obtain the final result. Each s_i is also a matrix with the same size as the input data u that in each location x depends only on $u_i(x)$ and $u(x)$ and then it can be calculated for all the locations x simultaneously (**step ii)**).

Finally, in **step i)** we show how the matrices u_i, which are defined as $u_i(x) = u(y_i, x)$, are obtained. The input data u is convolved with kernels $K_1, \ldots, K_9$ to obtain the $u_1, \ldots, u_9$ matrices. Each kernel K_i is a matrix that is everywhere '0' except in the $i-th$ position that stores a '1'. In order to keep the same width and height after each convolution zero padding is used, as it is usual in convolutional neural networks.

This implementation using convolutions allows us to extend the INRF-module for any input data size and any output size by just adapting the size of the kernel w. Let us first write (4) for the case in which the input data u has NCh_{in} channels:

$$\text{INRF}(x; u, w, \lambda, \sigma) = \sum_{c=0}^{NCh_{in}-1} \sum_{i \in I(x)} w_i^c \left[u_i^c(x) - \lambda \sigma \left(u_i^c(x) - u^c(x) \right) \right], \tag{6}$$

where the channel is indicated by the superscript c. Moreover, the output INRF can have NCh_{out} channels, so the INRF at position x becomes a vector of NCh_{out} components:

$$INRF(x) = \left[INRF^0(x), \ldots, INRF^d(x), \ldots, INRF^{NCh_{out}-1}(x) \right], \tag{7}$$

and each of these components is obtained as:

$$\text{INRF}^d(x; u, w, \lambda, \sigma) = \sum_{c=0}^{NCh_{in}-1} \sum_{i \in I(x)} w_i^{c.d} \left[u_i^c(x) - \lambda \sigma \left(u_i^c(x) - u^c(x) \right) \right], \tag{8}$$

This generalization for an arbitrary number of input and output channels is represented in figure 2, again for a 3×3 kernel. In fact, the kernel is implemented as $3 \cdot 3 = 9$ kernels of size $1 \times 1 \times NCh_{in} \times NCh_{out}$, where NCh_{in}, NCh_{out} are respectively the number of input and output channels.

2 Implementing INRF inside a convolutional neural network

2.1 INRF layer

A convolutional layer is a linear + nonlinear operation composed by a cross-correlation (convolution but without flipping the kernel w) plus a bias term evaluated in a nonlinear function, as seen for instance in[1]:

$$\sigma_2(w * x + b) \tag{9}$$

In equation 9 the operation $w * x$ is the cross-correlation (hereafter convolution following literature terminology) between a tensor of weights w and an input tensor x, b is a bias term, and σ_2 a nonlinear function (called activation function).

In this work, we replace all the operations inside σ_2 in equation 9 by our INRF-module (equation 2), that is:

$$\sigma_2\left(\sum_{i\varepsilon I(x)} w_i[u(y_i) - \lambda\sigma_1(u(y_i) - u(x))]\right) \tag{10}$$

This is what we call an INRF-layer, it has trainable weights w and it can fully replace a convolutional layer.

2.2 INRF neural networks

A convolutional neural network (CNN) is a stack of convolutional layers which usually ends in a multilayer perceptron (stack of fully connected layers), see Fig.3. In this setting, the stack of convolutional layer acts as a feature extractor that feeds a vector to the multilayer perceptron (classifier).

In a INRF neural network (INRFnet), instead of convolutional layers there are INRF layers. In a CNN it is common to find other kinds of layers between convolutional layers, such as: pooling, batch normalization[2], or dropout[3]. Our INRF layers can be used in combination with these other layers too.

2.3 Mapping between layers

An important feature of convolutional layers is their capacity to map between arbitrary dimensions. For instance, if an input x has dimensions $m \times n \times NCh_{in}$ a convolutional layer can map this to an output y with dimensions $m \times n \times NCh_{out}$, where NCh_{in} and NCh_{out} are not necessarily equal. An INRF layer is capable to perform this mapping too. This happens in what is referred as **step iii)** in figure 2, see the implementation details in the previous section.

3 Classification architectures

In this section two INRFnet architectures used for classification are described.

3.1 INRFnet2 - A 2-layer net for MNIST

INRFnet2 has input dimensions $28 \times 28 \times 1$, then two INRF layers with $\lambda = 1.1$ are used. The first INRF layer has 32 kernels of size 5×5 and the second one has 64 kernels of size 5×5. After each INRF layer there is a pooling layer with a 2×2 kernel. The result from the second pooling layer is passed to a fully connected layer with 500 nodes followed by a dropout layer (with 0.5 probability) that is also connected with a final fully connected layer of k nodes (one node per desired class). In both INRF layers, the nonlinearities σ_1 are rectified linear units (ReLU's) and σ_2 is just the identity.

3.2 INRFnet3 - A 3-layer net for CIFAR10, CIFAR100, SVHN

INRFnet3 has input dimensions $28 \times 28 \times 3$, then three INRF layers with $\lambda = 2$ are used. All the INRF layers have 192 kernels of size 5×5. After all the INRF layers a batch normalization layer is used followed by a pooling layer with 2×2 kernel in the first two layers. Finally, an average pooling layer (with kernel size of 8×8) is stacked after the third batch normalization layer. At the end a fully connected layer with k nodes (one node per desired class) is stacked. In all the INRF layers the nonlinearities σ_2 are all ReLU's and σ_2 is a non-simetrical activation function defined in the equation:

$$f(x) = \begin{cases} x^p & \text{if x} \geq 0 \\ -|x|^q & \text{otherwise} \end{cases},$$

where $p = 0.7$ and $q = 0.3$ for INRFnet3.

4 Training considerations for INRFnets.

The training procedure of an INRFnet is currently constrained by (1) computation time, (2) improvement techniques designed for CNNs, and (3) harder optimization. (1) Although our implementation of INRF is optimized to run in a GPU through a highly efficient automatic differentiation library (Pytorch[4]), it stays behind against the training time of a CNN of comparable size/parameters. This drawback is not a surprise since most of the optimization made in automatic differentiation libraries is achieved through convolution operations (which we already described as inefficient to describe INRF) and additional operations are required to implement INRF. (2) Techniques and empirical tricks such as batch normalization or preprocessing using whitening that have been developed through many years of research are designed for CNNs and not for networks with INRF modules. For instance, a preprocessing using whitening is known to improve generalization in CNNs, however, in INRFnet we found that this procedure leads to worse performance. We need then to test all these tricks and methods in order to find strong

regularization and improvement techniques. (3) INRF is a highly non-linear model, this feature means that INRF has a lot of representation capacity but also leads to a harder optimization. In particular, a CNN requires less regularization than an INRFnet equivalent with the same architecture/parameters. This harder optimization combined with constraint (2) makes the optimization of INRFnet a hard and sensitive procedure compared with a CNN even with few layers.

References

1. Goodfellow, I., Bengio, Y. & Courville, A. *Deep learning* (MIT press, 2016).

2. Ioffe, S. & Szegedy, C. Batch normalization: Accelerating deep network training by reducing internal covariate shift. *arXiv preprint arXiv:1502.03167* (2015).

3. Srivastava, N., Hinton, G., Krizhevsky, A., Sutskever, I. & Salakhutdinov, R. Dropout: a simple way to prevent neural networks from overfitting. *The journal machine learning research* **15**, 1929–1958 (2014).

4. Paszke, A. *et al.* Automatic differentiation in pytorch. (2017).

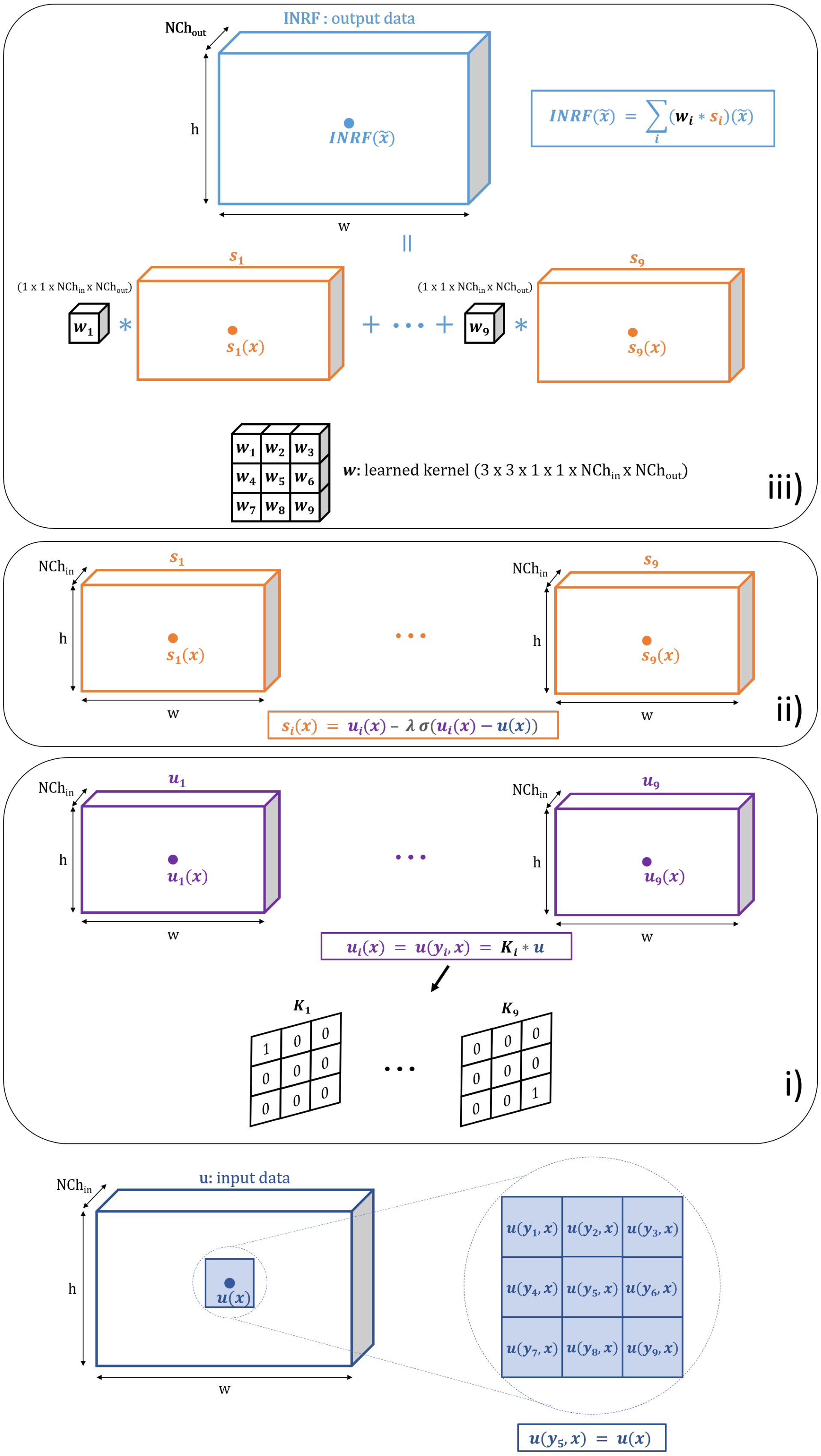

Figure 2. Implementation details of an INRF-module for any number of input and output data channels.

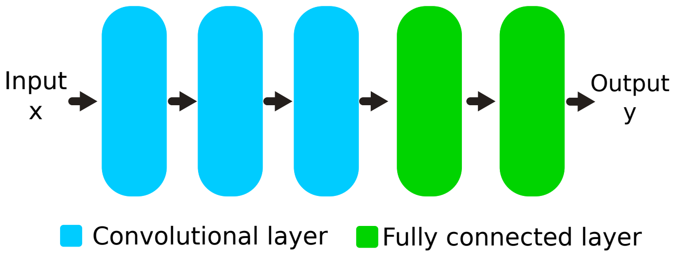

Figure 3. Convolutional neural network
